# Comparison of Safety and Efficacy of Anesthesia Methods in Percutaneous Endoscopic Lumbar Discectomy: A Network Meta-Analysis

**DOI:** 10.1155/prm/8022643

**Published:** 2024-12-06

**Authors:** Bin Zheng, Panfeng Yu, Yan Liang, Haiying Liu

**Affiliations:** Spine Surgery Department, Peking University People's Hospital, Beijing, China

**Keywords:** efficacy, epidural anesthesia, general anesthesia, local anesthesia, percutaneous endoscopic lumbar discectomy, safety

## Abstract

**Background:** The objective of this study was to systematically evaluate the safety and efficacy of local anesthesia, general anesthesia, and epidural anesthesia in percutaneous endoscopic lumbar discectomy (PELD).

**Methods:** We searched PubMed, EMBASE, and OVID databases for all relevant studies. All statistical analysis was performed using STATA 17.0.

**Results:** Fourteen studies were finally included, comprising 7 randomized controlled trials and 7 retrospective studies. The total number of subjects across these studies was 1655, with 316 undergoing general anesthesia, 789 undergoing local anesthesia, and 550 undergoing epidural anesthesia. The meta-analysis of pairwise comparisons suggests that there are no differences among epidural, general anesthesia, and local anesthesia in terms of postoperative VAS, ODI, and surgery time. Regarding complications, general anesthesia has a higher complication rate compared with local anesthesia, but there are no differences between epidural and general anesthesia or between epidural and local anesthesia. In terms of anesthesia satisfaction, both general and epidural anesthesia have higher satisfaction rates compared with local anesthesia, with no significant difference between general and epidural anesthesia. The ranking of the best probabilities shows that epidural anesthesia has the lowest postoperative VAS and highest anesthesia satisfaction. General anesthesia has the lowest ODI scores. Local anesthesia has the fewest complications and operative time.

**Conclusion:** Local anesthesia, general anesthesia, and epidural anesthesia are all safe and effective methods for PELD. Local anesthesia has advantages in complications and operation time. Epidural anesthesia is most advantageous in anesthesia satisfaction and postoperative VAS scores. General anesthesia is most advantageous in postoperative ODI. In the future, more multicenter RCTs are needed to further compare the safety and effectiveness of different anesthesia methods in PELD.

## 1. Introduction

Owing to the aging population and changes in lifestyle, the incidence of lumbar disc herniation is increasing. It has now become a major cause of low back pain [1, 2]. Although most patients experience pain relief through conservative treatments, some still require surgical intervention. Traditional open surgery is highly traumatic and risky and associated with many complications. Consequently, various percutaneous minimally invasive techniques have become the preferred option for many surgeons and patients, leading to rapid advancements in spine surgery [3, 4].

The basic approach of percutaneous endoscopic lumbar discectomy involves expanding soft tissues through a series of channels to create a perforated surgical path. Endoscopic technology allows the entire surgery to be visualized, thus reducing trauma and bleeding, increasing patient safety, and facilitating a quicker postoperative recovery [5–7].

Most percutaneous endoscopic lumbar discectomy (PELD) surgeries are performed under local anesthesia. This allows direct communication with the patient during surgery, enabling real-time feedback through lower limb activities to check for nerve damage, which enhances patient safety. Consequently, local anesthesia is the most commonly used anesthesia method. However, pain is frequently reported during spine endoscopy under local anesthesia, and some patients may even require surgery to be paused due to intolerable pain. In addition, patients' fear and anxiety may be heightened when they are awake during surgery, potentially affecting intraoperative hemodynamic stability [8–11].

To further improve the patient's surgical experience and safety, it is crucial to explore suitable anesthesia modalities for PELD. Current alternatives include general anesthesia and epidural anesthesia. The primary goal of choosing appropriate anesthesia is to ensure the safety and efficacy of the procedure [12]. Previous meta-analysis comparing epidural anesthesia and local anesthesia for PELD suggest that while epidural anesthesia offers more advantages in terms of intraoperative VAS, both methods are similar in terms of safety [13–15]. Mooney's meta-analysis comparing local anesthesia and general anesthesia in PELD indicates higher long-term efficacy and higher satisfactory rates but higher complication rates in general anesthesia [16]. Traditional meta-analyses have explored these methods but are limited to two-by-two comparisons and do not encompass multiple treatment comparisons. Therefore, comparisons among the three anesthesia methods have yet to be conducted.

Network meta-analysis, an extension of traditional meta-analysis, offers the advantage of evaluating the efficacy of multiple interventions simultaneously. In this study, we conducted a systematic review of the currently published literature and performed a network meta-analysis on the available evidence to compare local, general, and epidural anesthesia in laminectomy.

## 2. Methods

The present systematic review and meta-analysis were performed according to the preferred reporting items for systematic reviews and meta-analysis (PRISMA) guidelines. Besides, this study refers to the methodology and research format of previous study [17].

### 2.1. Search Strategy

The authors used the following search terms: “anesthesia” AND “percutaneous endoscopic lumbar discectomy or percutaneous transforaminal endoscopic lumbar discectomy or percutaneous interlaminar endoscopic discectomy” to search PubMed, Embase, and OVID for articles published up to May 2024. The reference lists of the included articles were also screened to avoid missing relevant studies.

### 2.2. Inclusion Criteria

The inclusion criteria, specified according to the PICOS framework, were as follows.

  P (patients): patients diagnosed with lumbar disc herniation or spinal stenosis.
  I (intervention): patients who underwent percutaneous endoscopic lumbar discectomy under epidural anesthesia or general anesthesia.
  C (comparison): patients who underwent percutaneous endoscopic lumbar discectomy under local anesthesia.
  O (outcomes): the outcomes included the postoperative VAS, ODI score, operative time, anesthesia satisfactory score, and incidence of complications.
  S (study design): all comparative studies: case–control studies, cohort studies, and randomized clinical trials. At least one of the above outcomes was required to be reported in the included studies. Only studies published in peer-reviewed journals were considered for inclusion. Only studies published in English were eligible for inclusion.

### 2.3. Exclusion Criteria

I. Patients diagnosed with tuberculosis, infection, scoliosis, fractures, tumors, or rheumatoid arthritis.
II. Studies or patients presented by other included articles, review studies, case reports, and technical notes.
III. Cervical surgeries.

### 2.4. Study Selection and Data Extraction

Two authors independently screened the literature by reading the titles, abstracts, and full texts and applying the inclusion and exclusion criteria. The corresponding author, Liu, supervised the entire process and resolved all discrepancies. The data extracted from the literature included (1) baseline data from the included studies, such as the first author and year of publication, country, study type, anesthesia method, and sample size; (2) information required for risk-of-bias assessment; and (3) outcome data, such as the postoperative VAS score, postoperative ODI, operation time, complications, and anesthesia satisfaction rate.

### 2.5. Evaluation of Risk of Bias

Only randomized controlled trials (RCTs) were eligible for risk-of-bias assessment. Following the PRISMA and Cochrane Collaboration criteria, two authors independently assessed the risk of bias in the included studies in the following areas: (1) random sequence generation; (2) allocation concealment; (3) blinding of participants and personnel; (4) blinding of outcome assessment; (5) incomplete outcome data; (6) selective reporting; and (7) other bias. For observational studies, the Newcastle–Ottawa Scale is applied to evaluate bias.

### 2.6. Data Analysis

Analysis was carried out via STATA 17 software. The NMA was conducted via the “network” package in Stata 15.0 according to the frequency framework. Continuous variables (VAS score, ODI score, and operative time) were analyzed via the mean difference (MD) and its 95% credible interval (CrI), whereas dichotomous variables (complications and satisfactory anesthesia) were analyzed via the odds ratio (OR). The relationships between interventions were presented via network plots, with each node representing an intervention. The size of a node was directly proportional to the sample size of each intervention. The edge between the two nodes indicated a direct comparison between the two interventions, and the thickness of each line was positively correlated with the number of randomized comparisons between the two corresponding interventions. The cumulative ranking curve (SUCRA) method was used to rank the relative effectiveness of each intervention. The surface under the cumulative ranking (SUCRA) curve method was used to rank interventions in terms of efficacy. The SUCRA values ranged from 0% to 100%. The closer the value was to 100%, the greater the likelihood that an intervention was among the most desirable treatments.

## 3. Results

**Figure 1:**
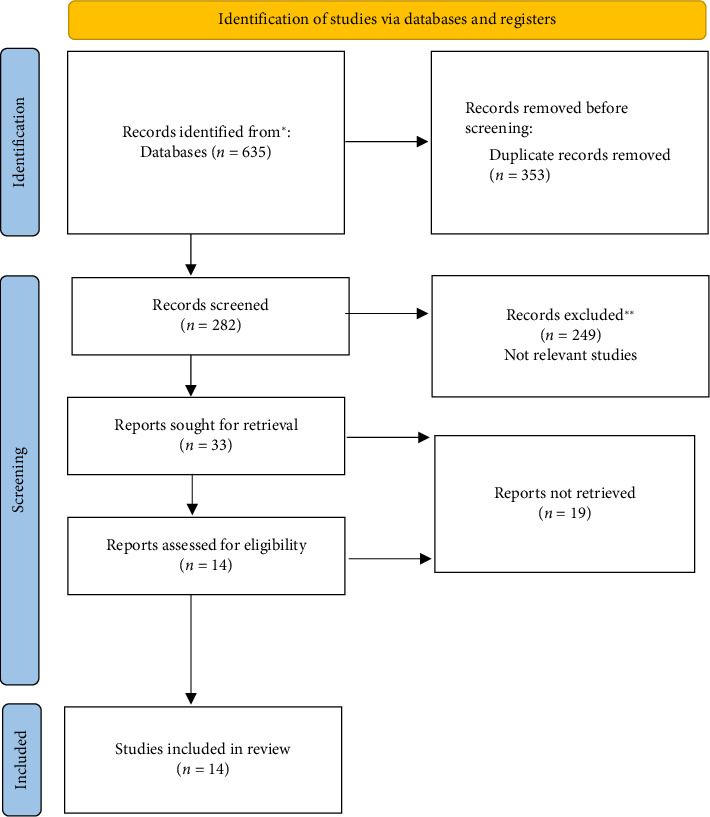
Flow diagram.

**Figure 2:**
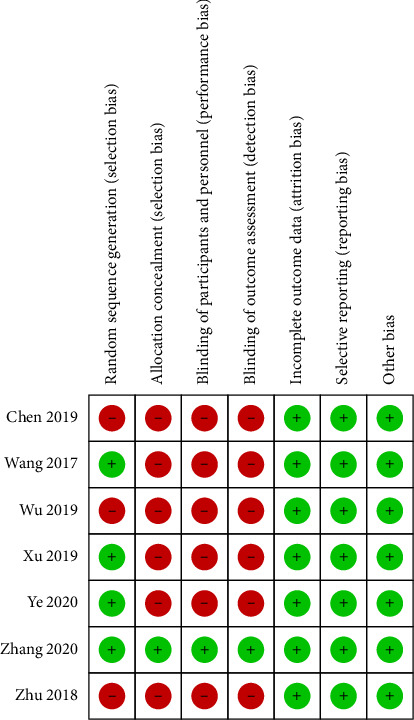
Risk of bias of randomized controlled trails.

Fourteen studies [8, 10, 18–29], comprising 7 randomized controlled trials [10, 18, 19, 23, 25, 27, 29] and 7 retrospective studies [8, 20, 22, 24, 26, 28, 29], were ultimately included. The total number of subjects across these studies was 1655, with 316 receiving general anesthesia, 789 receiving local anesthesia, and 550 receiving epidural anesthesia. The flowchart of the literature screening process is shown in Figure 1, and the results of the literature data extraction are presented in Table 1. The quality assessment of the RCTs via the Cochrane risk assessment tool is shown in Figure 2. Also, the risk of bias of observational studies is shown in Table 2.

### 3.1. Consistency Analysis

Overall consistency testing revealed good consistency between the direct and indirect comparisons. In addition, the node-splitting method was used for local inconsistency testing, which revealed no significant inconsistency. Therefore, a consistency model was used for the network meta-analysis.

### 3.2. Network Relationships

**Figure 3:**
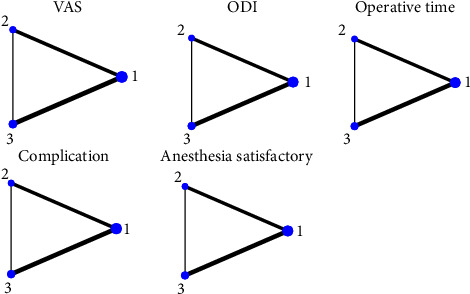
Network of anesthesia comparisons (Note: the width of the lines is proportional to the number of trials comparing every pair of treatments. The size of every circle is proportional to the sample size of interventions). (1) Local anesthesia. (2) General anesthesia. (3) Epidural anesthesia.

Figure 3 presents the network relationships between the postoperative VAS, postoperative ODI, surgery duration, complications, and anesthesia satisfaction. All of these forms form closed network relationships. Current comparisons are mainly between epidural anesthesia and local anesthesia, as well as between general anesthesia and local anesthesia. Fewer studies have compared epidural anesthesia and general anesthesia.

### 3.3. Meta-Analysis Results

#### 3.3.1. Postoperative VASs

**Figure 4:**
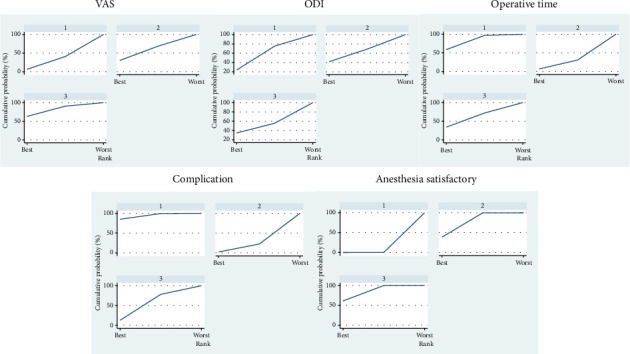
The surface under the cumulative ranking plots based on cumulative probabilities of interventions. (1) Local anesthesia. (2) General anesthesia. (3) Epidural anesthesia.

Eight studies [8, 10, 20, 23, 25, 26, 28, 29] were included, with three comparing general anesthesia vs. local anesthesia [8, 10, 20], four comparing epidural vs. local anesthesia [23, 25, 26, 29], and one comparing general vs. epidural anesthesia [28]. There was no difference between general and local anesthesia, no difference between epidural and general anesthesia, and no difference between epidural and local anesthesia, as shown in Table 3. The SUCRA ranking for the minimum VAS was epidural anesthesia (0.8) > general anesthesia (0.5) > local anesthesia (0.3), as shown in Table 4 and Figure 4.

#### 3.3.2. Postoperative ODI Scores

Seven studies were included [8, 10, 20, 23, 26, 28, 29], with three comparing general anesthesia vs. local anesthesia [8, 10, 20], three comparing epidural vs. local anesthesia [23, 26, 29], and one comparing general vs. epidural anesthesia [28]. There was no difference between general and local anesthesia, no difference between epidural and general anesthesia, and no difference between epidural and local anesthesia, as shown in Table 3. The SUCRA ranking for the minimum ODI values was as follows: general anesthesia (0.6) > local anesthesia (0.5) > epidural anesthesia (0.4), as shown in Table 4 and Figure 4.

#### 3.3.3. Operative Time

Twelve studies were included [8, 10, 18–23, 25–28], with seven comparing general anesthesia vs. local anesthesia [8, 10, 18–22], four comparing epidural vs. local anesthesia [23, 25–27], and one comparing general vs. epidural anesthesia [28]. There was no difference between general and local anesthesia, no difference between epidural and general anesthesia, and no difference between epidural and local anesthesia, as shown in Table 3. The SUCRA ranking for the minimum surgery duration was as follows: local anesthesia (0.8) > epidural anesthesia (0.6) > general anesthesia (0.2), as shown in Table 4 and Figure 4.

#### 3.3.4. Complications

Eleven studies were included [10, 18–24, 26, 28, 29], with six comparing general anesthesia vs. local anesthesia [10, 18–22], four comparing epidural vs. local anesthesia [23, 24, 26, 29], and one comparing general vs. epidural anesthesia [28]. Compared with local anesthesia, general anesthesia was associated with more complications; however, there was no difference in complications between epidural and local anesthesia, and there was no difference in complications between general anesthesia and epidural anesthesia, as shown in Table 3. The SUCRA ranking for the minimum number of complications was as follows: local anesthesia (0.9) > epidural anesthesia (0.5) > general anesthesia (0.1), as shown in Table 4 and Figure 4.

#### 3.3.5. Anesthesia Satisfaction

Seven studies were included [10, 18, 21, 23, 24, 26, 28], with three comparing general anesthesia vs. local anesthesia [10, 18, 21], three comparing epidural vs. local anesthesia [23, 24, 26], and one comparing general vs. epidural anesthesia [28]. Compared with local anesthesia, general anesthesia was associated with greater anesthesia satisfaction. Compared with local anesthesia, epidural anesthesia was associated with greater anesthesia satisfaction, and there was no difference in satisfaction between general anesthesia and epidural anesthesia, as shown in Table 3. The SUCRA ranking for maximum satisfaction was epidural anesthesia (0.8) > general anesthesia (0.7) > local anesthesia (0), as shown in Table 4 and Figure 4.

## 4. Discussion

Currently, local infiltration anesthesia is commonly used in PELD. Its advantages include convenience, low risk, low cost, and allowing patients to provide timely feedback during the operation, thus preventing accidental nerve root damage [30]. However, local anesthesia often provides insufficient pain relief, especially during adjustments of the puncture direction, handling of the zygapophyseal joints with a burr, dilating the working channel, and removing the nucleus pulposus, often causing patients to experience lower back pain or radiate leg pain, which affects their tolerance to the surgery. Additional opioid analgesics are frequently required during the procedure. This interrupts the surgeon's workflow, prolongs the operation, and can negatively affect the surgeon's performance. Intraoperative pain may also lead to increased heart rate and blood pressure, posing risks to patients with hypertension or coronary heart disease [7, 9]. General anesthesia is a common method that provides significant anesthetic effects but can easily damage patients' neurological functions [16]. Epidural anesthesia, which acts quickly and reliably, also allows for the extension of anesthesia duration and facilitates postoperative pain management while maintaining lower limb mobility. Low concentrations of ropivacaine can effectively prevent pain during surgery [13].

This study is the first network meta-analysis to assess the comparative effects and safety of three anesthetic methods during PELD surgery. The analysis indicated that general anesthesia is superior in terms of postoperative ODI scores, whereas local anesthesia has advantages in terms of complications and operation time. Epidural anesthesia has the best effect on postoperative VASs and anesthesia satisfactory.

The pairwise comparison results revealed no significant differences in postoperative VAS and ODI among the three anesthetic methods. Symptoms are caused primarily by herniated discs compressing the nerve roots. All three methods are effective in surgically removing herniated discs; thus, there are no differences in clinical outcomes after surgery. The VAS is used mainly for assessing pain intensity, whereas the ODI assesses patients' functional impairment and quality of life. Epidural anesthesia may be more advantageous for pain relief (epidural 80% > general 50% > local 30%), but general anesthesia is more beneficial for quality of life (generally 60% > local 50% > epidural 40%).

In terms of surgical efficiency, operation time is a useful parameter for assessing anesthetic effects. The pairwise comparison does not indicate significant differences, but some studies typically do not account for preparation time, anesthesia induction, or recovery, which could introduce discrepancies. My SCURA analysis revealed local anesthesia (80%) < epidural anesthesia (60%) < general anesthesia (60%). The results indicate that local anesthesia has advantages in terms of operation time that general anesthesia takes relatively longer and that epidural anesthesia requires procedures such as lumbar puncture and catheter placement, which extend the operation time [31]. In terms of overall complication rates, general anesthesia has a higher rate than local and epidural anesthesia does, with no difference between the latter two. SUCRA analysis of complication rates revealed that local anesthesia (90%) < epidural anesthesia (50%) < general anesthesia (10%), indicating that local anesthesia has an advantage in this area. Although general and epidural anesthesia have many advantages, they cannot completely replace local anesthesia. Local anesthesia remains the safe choice for most patients undergoing PELD, as timely pain feedback during surgery can effectively protect the integrity of nerve structures and prevent severe neurological damage.

In terms of anesthesia satisfaction, both general and epidural anesthesia are more satisfactory than local anesthesia is. Effective intraoperative analgesia is an important measure of anesthetic effectiveness; general anesthesia completely suppresses sensory and motor functions in the lower limbs. An appropriate concentration of epidural anesthesia can produce excellent sensory—motor blockade, effectively blocking sensory transmission and reducing intraoperative pain, thus enhancing the anesthesia experience [32, 33]. In the SUCRA analysis, local anesthesia did not achieve the highest level of satisfaction. Epidural anesthesia (80%) is more likely to provide a greater degree of anesthesia experience than general anesthesia (70%), possibly due to the increased complications associated with general anesthesia.

Based on the results of the meta-analysis, along with consideration of the patient's condition and underlying factors, we propose the following recommendations for the choice of anesthesia methods:

1. Safety of the procedure: local anesthesia has a lower rate of complications and a shorter operative time, making it suitable for patients aiming to minimize anesthesia-related risks.
2. Patient experience and comfort during surgery: (I) local anesthesia allows patients to provide real-time feedback during surgery, which helps avoid nerve structure damage, but it may cause discomfort and psychological stress during the procedure. It may not be ideal for patients with low pain tolerance. (II) Epidural anesthesia has the advantage of higher patient satisfaction by effectively relieving intraoperative pain and providing a good anesthesia experience. It is a good choice for patients who want to remain conscious but avoid pain. (III) General anesthesia completely eliminates sensations of pain and discomfort, making it suitable for patients who cannot tolerate intraoperative pain or experience significant psychological stress.
3. Special indicators: (I) local anesthesia is convenient for routine PELD procedures, enabling intraoperative monitoring and feedback. (II) For patients with cardiovascular conditions like hypertension or coronary heart disease, where discomfort may affect heart rate and blood pressure, epidural anesthesia offers stable hemodynamic effects. (III) For patients with low pain tolerance or concerns about intraoperative discomfort, general anesthesia is an effective choice, though it may carry a relatively higher risk of complications.

Limitations of this study: (1) first, the sample size is limited; this paper includes only 14 studies, which is not a large number. Only one paper compared epidural anesthesia with general anesthesia. (2) To ensure comprehensive analysis, this meta-analysis included all comparative studies. Seven of the 14 papers were RCTs, and the rest were retrospective studies. More multicenter RCTs are needed for evaluation safety and efficacy in different anesthesia methods. (3) Owing to limitations in the included literature, this study lacked an analysis of several important clinical indicators, such as hospital stay duration and hospitalization costs. In addition, owing to the characteristics of general anesthesia, assessing intraoperative pain scores is impractical. (4) Thirteen out of 14 studies are reported from China, lacking studies from other countries, which may not be applicable to patients from other countries. (5) Only studies published in English are included, lacking studies from other language.

## 5. Conclusion

Local anesthesia, general anesthesia, and epidural anesthesia are safe and effective methods for PELD. Local anesthesia has advantages in terms of complications and operation time. Epidural anesthesia is most advantageous in terms of postoperative VASs and anesthesia satisfactory. General anesthesia is most advantageous in terms of postoperative ODI. In the future, more multicenter RCTs are needed to further compare the safety and effectiveness of different anesthesia methods in PELD.

## Figures and Tables

**Table 1:** Charasteristics of the included studies.

Study	Study type	Country	Anesthesia method		Sample size	
Chen 2019	RCT	China	General anesthesia	Local anesthesia	50	73
Guan 2019	Retrospective study	China	General anesthesia	Local anesthesia	60	60
Wu 2019	RCT	China	General anesthesia	Local anesthesia	50	48
Ye 2020	RCT	China	General anesthesia	Local anesthesia	30	30
Kong 2021	Retrospective study	China	General anesthesia	Local anesthesia	29	26
Kerimbayev 2022	Retrospective study	Kazakhstan	General anesthesia	Local anesthesia	20	20
Wu 2023	Retrospective study	China	General anesthesia	Local anesthesia	36	72
Fang 2016	Retrospective study	China	Epidural anesthesia	Local anesthesia	165	121
Wang 2017	RCT	China	Epidural anesthesia	Local anesthesia	46	46
Zhu 2017	Retrospective study	China	Epidural anesthesia	Local anesthesia	65	67
Zhu 2018	RCT	China	Epidural anesthesia	Local anesthesia	80	80
Xu 2019	RCT	China	Epidural anesthesia	Local anesthesia	49	46
Zhang 2020	RCT	China	Epidural anesthesia	Local anesthesia	100	100
Ren 2020	Retrospective study	China	General anesthesia	Epidural anesthesia	41	45

**Table 2:** Risk-of-bias assessment using the Newcastle–Ottawa Scale for observational studies.

Study	Selection				Comparability		Outcome			Total scores
	Exposed cohort	Nonexposed cohort	Ascertainment of exposure	Outcome of interest	The most important factor	Additional factor	Assessment of outcomes	Length of follow-up	Adequacy of follow-up	
Guan 2019	★	★	★	★	★	★	★			7
Kong 2021	★	★	★	★	★	★	★		★	8
Kerimbayev 2022	★	★	★		★	★	★	★	★	8
Wu 2023	★	★	★		★	★	★	★	★	8
Fang 2016	★	★	★		★	★	★	★	★	8
Zhu 2017	★	★	★	★	★	★	★	★	★	9
Ren 2020	★	★	★	★	★	★	★	★	★	9

**Table 3:** The network meta-analysis results.

	1	2	3
*VAS*			
1	—	0.09 (−0.36–0.54)	0.22 (−0.18–0.62)
2	−0.09 (−0.54–0.36)	—	−0.18 (−0.62–0.26)
3	−0.22 (−0.62–0.18)	0.18 (−0.26–0.62)	—

*ODI*			
1	1	0.09 (−1.05–1.23)	−0.10 (−1.47–1.28)
2	−0.09 (−1.23–1.05)	2	0.9 (−1.72–3.52)
3	−0.9 (−3.52–1.72)	—	3

*Operative time*		1.88 (−1.22–4.98)	
1	—	−8.31 (−20.67–4.03)	−0.48 (−7.82–6.86)
2	8.31 (−4.03–20.67)	—	8.56 (−33.54 ± 50.67)
3	2.67 (−12.66–18.01)	0.48 (−6.86–7.82)	—

*Complication*			
1	—	—	—
2	1.17 (1.01–10.18)	—	1.76 (0.46–6.73)
3	1.90 (0.67–5.37)	—	—

*Anesthesia satisfactory*			
1	—	—	—
2	2.69 (1.43–5.05)	—	0.66 (0.14–3.15)
3	3.03 (1.82–5.10)	—	—

**Table 4:** Results of the surface under the cumulative ranking and probability.

Treatments/outcomes	SUCRA	PrBest	Mean rank
*Vas*			
Local anesthesia	0.3	6.5	2.5
General anesthesia	0.5	30.8	2.0
Epidural anesthesia	0.8	62.7	1.5

*ODI*			
Local anesthesia	0.5	21.4	2
General anesthesia	0.6	45.1	1.8
Epidural anesthesia	0.4	33.5	2.1

*Operative time*			
Local anesthesia	0.8	57.9	1.5
General anesthesia	0.2	5.8	2.6
Epidural anesthesia	0.6	36.3	1.9

*Complication*			
Local anesthesia	0.9	85.8	1.1
General anesthesia	0.1	3.1	2.8
Epidural anesthesia	0.5	11.1	2.1

*Anesthesia satisfactory*			
Local anesthesia	0	0	3
General anesthesia	0.7	37.3	1.6
Epidural anesthesia	0.8	62.7	1.4

## Data Availability

The data used to support the findings of this study are all included within this article and open to all readers.
